# Negative feedback circuit for toll like receptor-8 activation in human embryonic Kidney 293 using outer membrane vesicle delivered bi-specific siRNA

**DOI:** 10.1186/s12865-015-0109-9

**Published:** 2015-07-23

**Authors:** Anurag Adhikari, Birendra Prasad Gupta, Krishna Das Manandhar, Shravan Kumar Mishra, Hari Krishna Saiju, Rajendra Maan Shrestha, Nawneet Mishra, Shishir Sharma

**Affiliations:** Asian Institute of Technology & Management, Purbanchal University, Knowledge village, Khumaltar, Satdobato, Lalitpur, Nepal; Central Department of Biotechnology, Tribhuvan University, Kathmandu, Nepal; National Public Health Laboratory, Teku, Kathmandu, Nepal; Padma Kanya Multiple Campus, Tribhuvan University, Kathmandu, Nepal; South Asian University, New Delhi, India

**Keywords:** Bi-siRNA, TLR8, INF type I, Outer membrane vesicle, p19, Inflammation, Negative feedback

## Abstract

**Background:**

TLR8 assists in antiviral approach by producing Type 1 INF via MyD88 dependent IRF7 pathway. However, over expression of *INFα/β* molecule poses threat by developing tolerance in chronic infection cases and enhancing inflammatory response. Here we report a bi-specific siRNA based complex which differentially activates and silences the TLR8 and MYD88 respectively in a negatively regulated fashion.

**Results:**

Outer membrane vesicle from *Escherichia coli* used for siRNA delivery was observed more efficient when attached with invasive protein *Ail* along with *OmpA* (P < 0.001) in HEK293-TLR8 cell line. siRNA complexed with *p19* protein was efficient in activating TLR8, confirmed by the increment of *INFβ* molecules (P < 0.001) in HEK293-TLR8 compared to its counterpart. Fusion of lipid bilayer of endosomal compartment was significant at pH 4.5 when fusogenic peptides (*diINF-7*) were incubated in membrane vesicle, thus facilitating the escape of siRNA complex to the host cytoplasm in order to silence MyD88 transcript (P < 0.001).

**Conclusions:**

We investigated the activation of TLR8 by bi-specific si-RNA for the production of *INFβ*. In the same setting we showed that bi-specific si-RNA was able to silence MyD88 transcript in a delayed manner. For the cases of auto immune disease and inflammation where over activation of endosomal TLRs poses serious threat, bi specific siRNA could be used as negative feedback controlled system.

**Electronic supplementary material:**

The online version of this article (doi:10.1186/s12865-015-0109-9) contains supplementary material, which is available to authorized users.

## Background

Endosomal TLR7/8 are specialized for the detection of ssRNAs [1] and are component of innate immune response [2, 3]. TLR7/8 are activated by whole RNA from viruses and/or certain synthetic single-stranded oligoribonucleotides (ORNs) as well double-stranded small interfering RNA sequences [4, 5] among which, ssRNA strand of minimum 19 bp with high GU or U rich content are found to be more potent as TLR7/8 agonist [5]. Thus, TLR7/8 agonists are promising enough to be used as vaccine adjuvents [6] and in passive immunotherapy approaches [7].

The differential functionality of IFN Type I (IFN-I) produced after activation of TLR7/8 have been studied against broad range of viral infections. Interferon-α inducible protein 6 (IFI6) can inhibit the Hepatitis C virus (HCV) entry by inhibiting EGFR mediated CD81/CLDN1 interactions [8]. Similarly, in HBV infection, IFNα mediates suppression of enhancer region En I, which are critical for HBV gene expression and replication [9–11]. Also, in case of HIV-1 infection, the IFNα upregulated CD317(tetherin) inhibits the release of newly assembled virions [12, 13] whereas, tripartite-motif-containing 5α (TRIM5α) restricts the incoming HIV-1 capsid, which is also up regulated by Type 1 IFNs [14]. The role of IFN-I may always not be true as mentioned in above instances as in chronic cases like HIV/SIV infection. In these chronic cases, the pattern of IFN mediated antiviral response is dependent upon the amount and duration of IFN molecules administration [15]. The continued IFN treatment can induce IFN-I desensitization and decrease antiviral gene expression, enabling infection with increased SIV reservoir size and accelerated CD4 T-cell loss [15]. Even continued IFN production via TLR7/8 results in decreased Interferon Stimulating Gene (ISG) expression like cGAS, APOBEC3G, MX2, tetherin and TRIM5α in SIV infected models [15].

Hence, it seems crucial to delay the expression pattern of IFN in order to avoid the tolerance in those infected systems. This study focuses on the administration of siRNA, as agonist for TLR7/8, which initiates ISG expression formerly and simultaneously would silence the MyD88 transcript. The silencing happens only after escaping from the endosome, so as to halt the signaling between the TLR7/8 and ISG expression in MyD88-IRAK1-TRAF6-IRF7 cascade [16, 17]. Thus, activation of TLR7/8 inside endosome and the silencing of MyD88 mRNA in cytoplasm in a delayed timing is the main objective of this study.

## Methods

### Bacterial strain, plasmids and cell line

*Escherichia coli* (F- mcrA Δ (mrr-hsdRMS-mcrBC) φ80lacZΔM15 ΔlacX74 nupG recA1 araD139 Δ (ara-leu)7697 galE15 galK16 rpsL (Str^R^) endA1 λ^−^) was the model system used for the cloning and expression of the plasmids (Additional file 1) as well for the production of outer membrane vesicle for siRNA delivery. siRNA (Additional file 1) was prepared as described elsewhere [18]. In order to study the protein translocation, green fluorescence protein (*gfp*) was used as reporter system [19]. The pSB1C3 containing *E. coli* was induced using 20 μM arabinose and consecutively cells were washed and 1 mM IPTG was added to repress the pBAD derived promoter in pSB1C3 before fluorescence measurement. The relative fluorescence levels present in the different cellular fractions were quantified using Qubit®2.0 *Fluorometer* (485-nm excitation/535-nm emission). The human embryonic kidney cell line (HEK293) was stably transfected with pcDNA-TLR8-YFP (which was a kind gift from Doug Golenbock (Addgene plasmid # 13024) and was cultured in Dulbecco’s modified Eagle’s medium (DMEM), containing 4.5 g/l glucose and supplemented with 10 % (v/v) fetal bovine serum along with 50 U/ml penicillin, 50 mg/ml streptomycin, 200 μg/ml Neomycin and 2 mM L-glutamine.

### Outer membrane vesicle preparation and invasion assay

*E coli* overnight culture having 0.8 OD_600_ were subjected for centrifugation at 6,000 × g and supernatant was filtered through *Durapore*® sterilizing-grade hydrophilic polyvinylidene fluoride (PVDF) membranes (Merck Millipore). The OMVs were collected after ultracentrifugation at 100,000 × g at 4 °C (Optima™ XE, Beckman coulter) for 3 h and finally resuspended in PBS [20]. To determine the endocytosis of OMVs in HEK293-TLR8 cell line, the *Ail* [21] along with *OmpA* [22] were expressed in *E coli* using pSB1C3. The invasive activity was determined on the basis of *Ail.OmpA* mediated endocytosis and escape from the late endosome provoked by fusogenic peptide, *diINF-7*. The invasion assay was done for 60 minutes allowing co-incubation of purified OMVs and HEK293-TLR8 cell. The incubation was followed by washing of cells to remove attached OMVs on surface and cell preparation for further analysis.

### *p19*.siRNA complex dissociation

Dissociation of siRNA from the *p19*.siRNA complexes was observed by gel electrophoresis. The outer membrane vesicle containing pSB1C3 construct for *p19*.siRNA.*torA* were subjected to lysis in GES reagent (100 mM EDTA pH8.0, 5 M guanidinium thiocyanate, and 1 % (w/v) N-Lauroylsarcosine salt of sodium) then were vortexed for 20s to allow the homogenization. The lysed content were incubated in the pH conditions ranging from 7.5 to 5.0 for 30 min at room temperature then each sample were electrophoresed on 20 % polyacrylamide gel. The composition for each pH were as follows: pH = 7.5 (PBS buffer (7.5), pH = 7.0 (200 mM dibasic sodium phosphate and 100 mM citric acid), pH = 6.5 (200 mM monosodium phosphate and 200 mM disodium hydrogen phosphate), pH = 6.0 (100 mM citric acid and 100 mM sodium citrate), pH = 5.5 (20 mM sodium acetate buffer, 100 mM sodium chloride), and pH = 5.0 (200 mM sodium cacodylate and 200 mM hydrogen chloride).

### Cytotoxicity assay for outer membrane derived vesicles

HEK293-TLR8 cells were seeded on a 96-well plate at a cell density of 1 × 10^4^ cells/mL and was grown for 24 h in DMEM with 10 % FBS. Cells were then co cultured with all available OMVs (Additional file 1) complexes per well (50 μl OMV [OD_600_ 0.8]/well). Fifty microliter of CytoTox-Glo™ Cytotoxicity Assay Reagent (Promega, USA) per well (12.5 μl for a 384-well plate) of HEK293-TLR8 was also added and luminescence was measured after incubating for 15 minutes at room temperature. After that, 50 μl of lysis reagent was added to all wells (12.5 μl for a 384-well plate), mixed and incubated at room temperature for 15 minutes. Then again, luminescence was measured and the viable cell luminescence was calculated as manufacturer’s instruction.

### Enzyme Link Immunosorbent Assay for *INFβ* quantification

ELISA for *IFN-β* was done in every 2 hours using different confluence state of HEK-TLR8 (70 %, 80 %, 90 %) each after 60 minutes of OMVs invasion assay using Human *IFN-β* ELISA kit (Tocris Bioscience, MN, USA) and was performed according to manufactures instruction.

### MyD88 gene silencing in vitro

In vitro gene silencing efficacy of siRNA complexes was evaluated with MyD88 gene expressing HEK293-TLR8 cells. We performed the reverse transcription-polymerase chain reaction (RT-PCR) to analyze the MyD88 silencing efficacy of siRNA complexes. RT-PCR was performed using the same volume of cultured cell in triplicate but of different confluence state (70 %, 80 %, 90 %), and 60 minutes before RT-PCR, the cell line was transfected with OMV aliquot of same concentration (50 μl OMV [OD_600_ 0.8]/well) and this process was repeated until 48 hr of cell growth cycle. Every time, the cells were harvested, lysed, and total RNAs were extracted using TRIzol® Reagent (Thermo-Fisher, USA) and reverse transcription was performed using one step RT-PCR kit (QIAGEN, Germany) according to the manufacturer’s protocol in triplicates. The PCR primers (Additional file 1) were synthesized and purified by IDT (California, USA). The quantitative analysis was done using Rotor-Gene 6000 (Corbett Life Science, Valencia, CA, USA).

### Statistical analyses

Results were analyzed using GraphPad Prism 6.0 software (GraphPad Software Inc, La Jolla, CA, USA) and the results were analyzed by Student’s t test which is parametric and p value < 0.001 was considered as significant result, unless described otherwise.

## Results

### Transport of torA-fusion proteins to periplasm of *Escherichia coli*

**Fig. 1:**
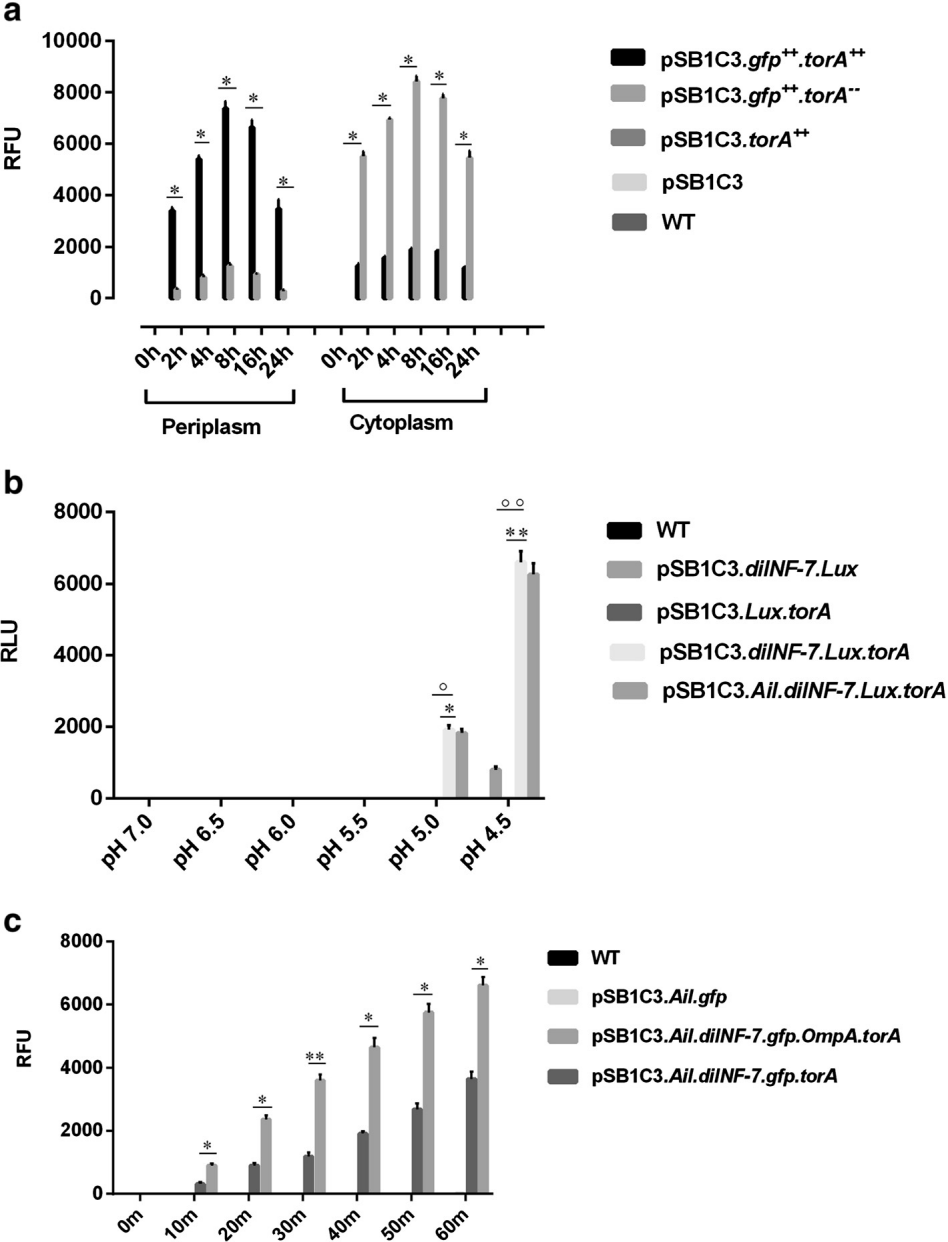
**a** Transport into outer membrane vesicle: In the presence of periplasmic signal (*torA*) *gfp* transportation to periplasm was significant (*P < 0.001) compared to construct which lacked *torA* signal (n = 10). **b** Fusion of lipid bilayer by *diINF-7*: The fusogenic activity was observed in presence of *diINF-7* along with *torA* signal at pH 5.0(*P < 0.001) and 4.5(**P < 0.001). Also, the fusogenic activity was affected by presence or absence of *torA* signal peptide at pH 5.0(°P < 0.001) and 4.5(°°P < 0.001) but was independent of presence or absence of *Ail/OmpA*. **c** TLR8-HEK293 invasion assay: In presence of *OmpA* signal, the invasion was rapid as well at 30 minutes of assay the fluorometric output increased significantly (**P < 0.001). Values show the mean ± S.D (n = 10). The statistical analysis was performed by unpaired Student’s t test (*P < 0.001)

In order to transport the siRNA, we needed siRNA to be packed in outer membrane vesicle (OMVs), thus we devised torA fusion system which would efficiently transport the proteins from cytoplasm to periplasm of *E.coli.* The relative light unit for OMVs quantitatively increased in first 8 hr from cells containing *torA* fused gfp in pSB1C3 backbone (P < 0.001) but the vesicle without *torA* fusion backbone failed to generate significant fluorescence. In the absence of periplasmic signal peptide construct *torA,gfp* wasn’t transported up to periplasm from cytoplasm in order to emit florescence (Fig. 1a). In this way, all the siRNA and accessory proteins were packed in OMVs and were ready for transfection in HEK293-TLR8 cell line.

### Ail mediated endocytosis of outer membrane vesicle in HEK293-TLR8 cell line

After efficient packaging of TLR8 agonist and MyD88 silencing components in OMV, we asked how efficient this system is in entering the HEK293-TLR8. Delivery of cargo vesicle inside host cell was found to be increased significantly with the use of invasive protein *Ail* from *Yersinia pestis*. The fluorometric analysis done pre/post OMV invasion in confluent (90 %) HEK-293-TLR8 cell line confirmed the increament in intracellular fluorescence for the OMVs from pSB1C3.*Ail.diINF-7.gfp.torA.* Further, there was >2 fold increments observed in RFU unit on the OMVs from pSB1C3 which had 464 bp of *OmpA* attached at the NH_2_ terminal of *Ail* in its 60 minutes assay (Fig. 1c). The co-incubation of purified OMVs and HEK293-TLR8 cell followed by washing of cells and observation of fluorometric output in every 10 minutes showed the increment up to 30-40 % (P < 0.001) of maximum RFU value within 20 minutes of the assay, suggesting a rapid invasion of OMVs attached with *OmpA* (pSB1C3.*Ail.diINF.gfp.OmpA.torA*) but not for OMVs from pSB1C3.*Ail.diINF.gfp.torA*. The latter had 30-40 % of maximum RLU after 30 minutes, suggesting slower HEK293-TLR8 invasion and endosomal escape in absence of *OmpA* (Fig. 1c). Treatment with colchicine had shown little effect in invasion as shown by reduced (2–3 fold for 8 μg/ml) fluorometric value of pSB1C3.*Ail.diINF-7.gfp.OmpA.torA* and pSB1C3.*Ail.diINF-7.gfp.torA,* suggesting the invasion in fact is mediated by cytoskeletal movements (data not shown).

### Fusogenic activity of diINF-7 in outer membrane vesicles

In the line of our hypothesis, pH dependent fusion of lipid bilayer was achieved in *E. coli* derived OMVs containing *diINF-7* peptide fragment. The OMV cargo which included the agonist for TLR8 should be cut open in order to free the components so it can act as ligand for TLR8 and as well escape the endosome so as to arrive in cytoplasm. The construct pSB1C3.*diINF-7.lux.torA* in an IPTG repressible promoter pBAD were transfected in *E.coli*, and OMV vesicles were isolated in order to measure the luminescence value at different pH buffer. The vesicle broke down at 4.5 pH thus providing highest RLU value (P < 0.001), irrespective of presence of *Ail/OmpA* (Fig. 1b).

### Escape from endosomal cargo to cytoplasm

**Fig. 2:**
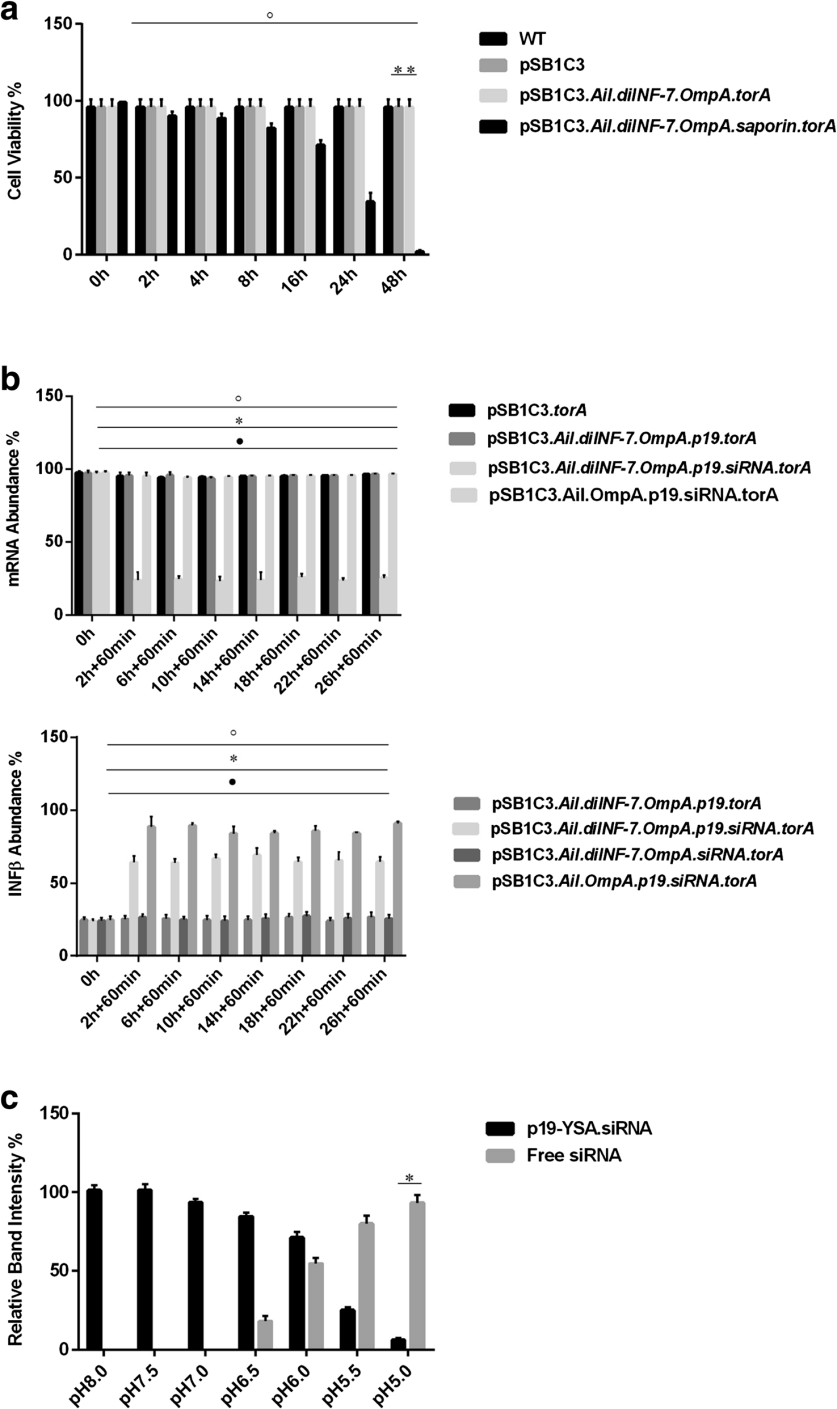
**a** Cell death as marker of transport to cytoplasm: The cell viability decreased in presence of saporin which peaked in 48 h (**P < 0.001). The construct lacking any of *Ail *(°P < 0.001), *diINF-7* (°P < 0.001), *torA * (°P < 0.001) and *OmpA* (°P < 0.001) failed to decrease the cell viability significantly in presence of *saporin* construct. **b** The presence of *p19 *was essential for binding of siRNA (°P < 0.001). The addition or deletion of *p19 * (°P < 0.001)/*siRNA* (*P < 0.001)/*diINF-7* (•P < 0.001) had significant effect in *INFβ * production as well MyD88 transcript abundance. **c** pH dependent dissociation was observed and siRNA was free from *p19* complex (*P < 0.001). Values show the mean ± S.D (n = 10). The statistical analysis was performed by unpaired Student’s t test (*P < 0.001)

Since we knew in vitro that silencing and agonist cargo could be escaped from OMVs at pH 4.5 but could it be used to escape endosome as well. The answer was yes, and to demonstrate the phenomenon we used saporin so as to mark the escape of endosomal cargo to cytoplasm. The cell death was used as a indicator for endosomal escape since saporin is a ribosomal inactivating protein. The OMVs derived from construct pSB1C3.*Ail.diINF-7.OmpA.saporin.torA* was able to decrease the cell survival percentage significantly after 16 hr of co culture. After 48 hr of co culture, the cell growth completely stopped and cell mortality was near 100 %. Construct lacking at least any of *saporin/diINF-7/Ail/OmpA/torA* was unable to initiate cell death (Fig. 2a).

### siRNA binding protein complex p19 for efficient transportation

To avoid siRNA degradation and efficient delivery, we used *p19* siRNA Binding Protein (19 kDa) from the Carnation Italian Ringspot Virus (CIRV) plant which binds siRNAs with nanomolar affinity. After confirmation of cargo transport up to cytoplasm, we deployed OMVs equipped with 21ntd. siRNA derived from the pSB1C3.*Ail.diINF-7.OmpA.p19-siRNA.torA* construct. The silencing of MyD88 as well as *IFNβ* was observed after 2 hr of co culture, and also there was a significant difference in the activity in presence and absence of *p19* specifically between 10–12 hr of co culture (Fig. 2b). This showed that, siRNA can be packed in with *p19* which would transport and save it from nucleases in host cytoplasm.

### pH dependent siRNA dissociation from transporter complex *p19*

We confirmed that siRNA could be detached from *p19* complex in a pH dependent manner so p19 couldn’t interfere in siRNA’s functional activity. The OMVs (derived from pSB1C3.*Ail.diINF-7.p19-siRNA.torA*) treated with decreasing pH conditions and simultaneous extracts from the buffer showed high intensity at low pH in polyacrylamide gel. The polyacrylamide gel assay showed the optimum dissociation of siRNA-*p19* complex at pH 5.0 with maximum relative band intensity compared to pH 7.0, 6.5, 6.0, 5.5 (Fig. 2c). The result showed that, at increasing acidic condition of endosomal compartment, the siRNA-*p19* complex might dissociate and thus facilitate siRNA binding to TLR8 as ligand and as well escape to cytoplasm for the formation of RISC complex.

### siRNA as ligand for TLR8 & regulator for *IFNβ* production by MyD88 silencing

The *IFNβ* production as well as rate of silencing of MyD88 was significant in presence of siRNA (P < 0.001). There was a fluctuating plane for the *IFNβ* production in presence of siRNA construct providing the efficient silencing of mRNA in a delayed timing (Fig. 2b). The transcript of MyD88 diminished by >4 folds when the OMVs construct along with siRNA complex was incubated with HEK293-TLR8 (Fig. 2b). As expected the OMVs construct lacking at least any of *Ail/diINF-7/siRNA/torA* from pSB1C3.*Ail.diINF-7.OmpA.siRNA.torA* didn’t show any decrement on the MyD88 mRNA. *IFNβ* production decreased as much as by 28 % from the construct siRNA.*diINF-7* compared to siRNA alone, due to the silencing the adaptor protein MyD88’s transcript (Fig. 2b).

## Discussion

We developed a mechanism which combines siRNA production, delivery, attachment to target cell, endocytosis, activation of TLR-8 and final escape from endosomal compartment to silence MyD88 transcript. The result of this study showed that, bi specific siRNA can be used differentially to activate TLR8 as ligand and silence MyD88 transcript, thus making a temporary stop at the over activation of TLR8 which can otherwise evokes inflammatory cascade. TLRs are important component of innate immune system which works for virus inhibition, DC maturation to antigen uptake for CD4^+^ T helper cell differentiation based on the activation of different cytokines expressed under influence of ISGs [22–24]. Cytokines induce positive feedback in immune system but if left unchecked can cause immunopathology like Crohn’s and inflammatory bowel disease, which are results of unbalanced immune response [25]. Duration of TLR mediated response is controlled by different factors such as, number of TLR expressed, availability of ligands and competitive features of ligand. Termination of TLR signals by regulation of receptor imminent molecules are described for different proteins; however, components that restrict MyD88 transcript are poorly defined specifically in case where initially the same component would activate IRF7 cascade [26–29].

In case of HIV-1 infection, TLR8 recognizes HIV-1 ssRNA during HIV-1 infection, where HIV-1 uses TLR8 signaling to activate NF-Κb for production of Tat-Rev mRNA via MyD88 dependent cascade [30] in dentritic cell but in the same setting when MyD88 was silenced, the replication of HIV-1 was aborted thus suggesting NF-κB activation by TLR8 triggering is an absolute prerequisite for HIV-1 transcription initiation by RNAPII. Thus, using bi-siRNA as described in our work, the initial antiviral cytokines could be expressed and later by silencing MyD88 in differential manner; the transcription of viral mRNA could be halted.

Since, DC infection is primarily involved in HIV-1 transmission via sexual act, inhibitors of MyD88 signaling cascade might represent as new antiretroviral drug although escalating the innate system via inflammatory cytokines in first place is immense due to its anti viral properties.

Constitutive MyD88 dependent TLR signals are proved to be advantageous, inflammation devoid signals [31]; however, these also could trigger inflammation, thus question on how to differentiate between inflammatory and homeostatic TLR signals remains. Proteins like IRAK-M, SIGIRR and suppressor of cytokine signaling (SOCS-1) can restrict TLR homeostatic signals from becoming inflammatory by restricting the duration and intensity of TLR signals [32,33]. MyD88 is an crucial signal transducer in *Helicobactor pylori* infected epithelial cells of human [34] which can induce proinflammatory cytokines including IL-6, IL-10, IL-1β and IL-12 in a TLR dependent manner [35,36]. It was reported that, RNA interference by miR-155 is able to negatively regulated the release of proinflammatory cytokines in *H. pylori* infected systems [37,38]. We show here that, siRNA exerts critical negative regulatory role thus lowering the *IFNβ* production. Consistent with the hypothesis, OMVs construct containing siRNA had decreased *INFβ* production, dependent upon the OMVs concentration thus providing a temporary stopover in TLR signaling (Fig. 2b).

In cardiac cells, MyD88 proteins are ubiquioustly expressed in cytoplasm but only after the signaling triggered by viral ssRNA, rapid redistribution of cytoplasmic MyD88 is seen in endoplasmic compartment [39] suggesting a time interval between recognition of ssRNA and MyD88 recruitment. Also, in another study, expression of TLR8 was significantly increased in case of ulcerative colitis (350 folds) and Crohn’s disease (45 folds) thus suggesting role of inflammatory cytokines like TNFα in inflammatory bowel disease (IBD) [40] which could be fatal and in these clinical cases, if temporary halt is to be made then partial inhibition of MyD88 is needed. Thus, initial activation of anti viral components and later inhibiting the inflammatory process may be useful in patients with inflammatory disease who are also infected with virus. The delivery of siRNA using OMVs could pave a novel way for future drug delivery options though the stability of siRNA remains a hurdle as well. Usage of MyD88 silencing can have negative impact on the activation of other TLRs beside TLR-8, which could pose a limitation in this approach. Interestingly, since we have used human embryonic kidney cell line, the apical TLR signal might preferentially induce homeostatic signal in this cell type, but in case of dendritic cell, it could have triggered inflammation. So the nature of activation is dependent in cell type as well. Another question raised by this study is how to determine the exact value of TLR8 activation and MyD88 transcriptional deactivation as well as its kinetics which would answer, for how long the activation and silencing would be beneficial to certain diseased state. This issue could only be addressed, when the experiment is carried out in vivo with subjected diseased state, where the dose dependent outcome could be monitored, which is also a limitation of this study.

This study supports the notion that MyD88 signals are to be maintained at threshold for proper homeostasis in innate system. However, above that threshold, innate inflammatory responses may be evoked which may result in chronic inflammation. But, also, insufficient MyD88 signalling would result in impaired situations for host survival, including, antimicrobial peptide secretion and tissue repair from acute inflammatory response. The finding of this study should be elaborated in future for distinguishing the needed degree of activation of TLR and deactivation of MyD88 thus avoiding inflammatory responses.

## Conclusion

The study devises a mechanism to pack and deliver endogenously expressed bi specific siRNA which could be used as TLR8 agonist as well as to silence MyD88 transcript. This mechanism could be used in case of inflammatory disease with viral infection where a negative component is needed to avoid inflammation and in the same time innate system should be activated to avoid foreign intrusion. Also, the system is interesting enough to be used in primary cell lines to establish new set of data on silencing and activating TLR8.

## Additional file

Additional file 1:
**Plasmid cassettes cloned in TOP10** ***Escherichia coli,*** **siRNA design and Outer membrane vesicles.** (RTF 171 kb)

## Abbreviations

TLR: Toll like receptor
INF-I: Interferon Type I
INFβ: Interferon beta
MyD88: Myleoid differentiating factor 88
EGFR: Epithelial Growth Factor Receptor
siRNA: silencing RNA
ssRNA: single stranded RNA
CTL: Cytotoxic T lymphocyte
GFP: Green florescence protein
Lux: Luciferase gene expression cassette
IPTG: Isopropyl β-D-1-thiogalactopyranoside
RLU: Relative luminescence unit
RFU: Relative florescence unit
IRAK-M: IL-1R–associated kinase M
SIGIRR: Single Ig and TIR domain
