# Supplementary material for: Negative feedback circuit for toll like receptor-8 activation in human embryonic Kidney 293 using outer membrane vesicle delivered bi-specific siRNA
Source: BMC Immunol. 2015 Jul 23;16:42. doi: 10.1186/s12865-015-0109-9 (PMC4510891; doi:10.1186/s12865-015-0109-9)
Supplement: Additional file 1: — Plasmid cassettes cloned in TOP10 Escherichia coli, siRNA design and Outer membrane vesicles. (RTF 171 kb) [file 12865_2015_109_MOESM1_ESM.rtf]

1.	DNA Parts used
·	torA
·	diINF-7
·	Ail
·	OmpA
·	Saporin
·	shRNA
·	p19
·	gfp
Sequence of parts used:
1. RBS+torA+diINF-7 with flanking site for restriction enzymes as given below

site for EcorI+XbaI-------gaattcgcggccgcttctagag                                                                                            taaggctcacggcgataagaaggaagaaaaataatgaacaataacgatctctttcaggcatcacgtcggcgttttctggcacaactcggcggcttaaccgtcgccgggatgctggggccgtcattgttaacgccgcgacgtgcgactgcggcgcaagcggcgactgacgctgtcatctcgaaagagggcattGGCTTATTTG AGGCTATCGA AGGTTTTATC GAGAATGGCT GGGAAGGTAT GATCGATGGC TGGTATGGGT GC ------- site for SpeI+PstI - tactagtagcggccgctgcag

2. Saporin with flanking site for restriction enzymes as given below as well gblock 20bp overhang

gBlock 1:

 site for EcorI+XbaI------ATGAAAATCTACGTCGTGGCCACCATCGCCTGGATCTTGTTGCAGTTTAGCGCCTGGACCACGACTGACGCCGTGACGTCCATTACCCTGGATCTGGTAAATCCGACAGCGGGCCAATATTCTAGCTTCGTGGATAAGATTCGCAACAACGTAAAAGATCCGAATCTGAAGTATGGTGGGACAGATATTGCCGTCATTGGTCCGCCGTCAAAAGAAAAGTTCTTACGTATTAATTTTCAATCAAGCCGCGGGACCGTGTCCTTGGGTCTCAAACGTGATAATTTATACGTGGTGGCGTACCTCGCAATGGATAACACCAATGTCAATCGTGCTTATTATTTCCGTTCTGAGATTACTTCCGCCGAACTGACCGCGCTGTTTCCTGAAGCGACTACCGCAAATCAGAAGGCGCTGGAGTACACCGAGGATTATCAAAGTATTGA -------20bp overhang to get gibson assembly with another gblock fragment

gBlock 2:
 
-------20bp overhang to get gibson assembly with another gblock fragment---GGAGTACACCGAGGATTATCAAAGTATTGAAAAAAACGCCCAGATCACGCAGGGCGATCAATCCCGTAAAGAATTGGGGCTGGGGATTGATCTGCTCTCGACGAGCATGGAGGCGGTAAACAAAAAAGCTCGCGTGGTTAAAGATGAAGCCCGTTTTCTGCTGATTGCAATCCAAATGACCGCAGAGGCTGCCCGCTTTCGTTACATCCAAAATCTGGTTATCAAAAATTTCCCAAACAAATTTAATTCTGAAAATAAAGTCATTCAATTCGAAGTAAATTGGAAAAAAATCTCGACGGCCATTTACGGAGACGCAAAAAACGGTGTGTTTAACAAAGACTATGACTTCGGTTTTGGCAAAGTTCGCCAAGTAAAAGATCTGCAGATGGGCCTGCTGATGTATCTGGGGAAACCAAAATCTTCCAACGAAGCTAATTCTACCGCGTACGCGACCACCGTCCTG
 ----- site for SpeI+PstI

3. p19+shRNA
 site for EcorI+XbaI------ATGGAGCGGGCGATCCAAGGCAACGATACACGCGAGCAGGCCAACGGTGAGCGTTGGGATGGGGGTTCAGGAGGCATCACGTCTCCGTTCAAACTGCCAGACGAATCGCCTAGCTGGACGGAATGGCGCCTGTACAATGATGAGACCAATTCGAACCAGGACAATCCCCTGGGCTTCAAGGAAAGCTGGGGCTTTGGAAAAGTCGTCTTCAAACGCTACTTGCGCTACGATCGCACCGAAGCCTCGCTGCATCGCGTGCTGGGTTCTTGGACCGGTGACAGTGTGAACTACGCTGCAAGTCGCTTCCTTGGTGCTAACCAGGTGGGTTGTACCTATTCTATTCGCTTCCGCGGTGTTAGCGTGACCATCAGTGGGGGCAGCCGCACACTGCAACACCTGTGCGAAATGGCCATTCGGAGTAAACAGGAGTTGTTGCAATTGACGCCGGTAGAAGTCGAAAGCAATGTTAGTCGTGGCTGCCCGGAGGGTATTGAGACTTTCAAAAAGGAATCAGAA
 ----- site for SpeI+PstI

4. Ail

 site for EcorI+XbaI------ ATGAAGAAAACACTGTTGACGTCTTCACTTATCGCGTGCCTGTCAATTGCATCGGTGAATGTTTATGCCGCATCAGAAAACTCGATTAGCATTGGTTACGCCCAATCCCATGTCAAGGAGAACCGTTACACCCTCGATAATGACCCAAAGGGCTTCAACCTGAAGTACCGCTACGAACTGGATGATAATTGGGGCGTGATTGGTAGTTTTGCCTATACGCATCAAGGTTATGATTTTTTCTATGGGTCTAACAAATTCGGCCATGGCGACCTCGATTATTACTCTGTTACGATGGGTCCGTCATTTCGCATCAACGAATACGTATCGTTATACGGTCTTCTTGGCGCCGCACATGGAAAAGTGAAATCCTCGGTCTTTGATGGTTCTGTGTCCACTAGCAAAACGTCGATGGCCTATGGCGCGGGCGTACAGTTTAACCCGCTTCCGAATTTTGTAATTGACGCGTCGTACGAGTACTCGAAACTGGATTCCGTTAAGTTCGGCACCTGGATGCTGGGCGCAGGATACCGCTTT
 ----- site for SpeI+PstI

5. Gfp in plasmid backbone pSB1C3

>BBa_E0040 Part-only sequence (720 bp)
Atgcgtaaaggagaagaacttttcactggagttgtcccaattcttgttgaattagatggtgatgttaatgggcacaaattttctgtcagtggagagggtgaaggtgatgcaacatacggaaaacttacccttaaatttatttgcactactggaaaactacctgttccatggccaacacttgtcactactttcggttatggtgttcaatgctttgcgagatacccagatcatatgaaacagcatgactttttcaagagtgccatgcccgaaggttatgtacaggaaagaactatatttttcaaagatgacgggaactacaagacacgtgctgaagtcaagtttgaaggtgatacccttgttaatagaatcgagttaaaaggtattgattttaaagaagatggaaacattcttggacacaaattggaatacaactataactcacacaatgtatacatcatggcagacaaacaaaagaatggaatcaaagttaacttcaaaattagacacaacattgaagatggaagcgttcaactagcagaccattatcaacaaaatactccaattggcgatggccctgtccttttaccagacaaccattacctgtccacacaatctgccctttcgaaagatcccaacgaaaagagagaccacatggtccttcttgagtttgtaacagctgctgggattacacatggcatggatgaactatacaaataataa

6. OmpA in plasmid backbone pSB1C3
>BBa_K103006 Part-only sequence (464 bp)
catatgaaagctactaaactggtactgggcgcggtaatcctgggttctactctgctggcaggttgctccagcaacgctaaaatcgatcagggaattaacccgtatgttggctttgaaatgggttacgactggttaggtcgtatgccgtacaaaggcagcgttgaaaacggtgcatacaaagctcagggcgttcaactgaccgctaaactgggttacccaatcactgacgacctggacatctacactcgtctgggtggcatggtatggcgtgcagacactaaatccaacgtttatggtaaaaaccacgacaccggcgtttctccggtcttcgctggcggtgttgagtacgcgatcactcctgaaatcgctacccgtctggaataccagtggaccaacaacatcggtgacgcacacaccatcggcactcgtccggacaacggcggaggttctggaggagggagctc


2.	Plasmids
S.N	Plasmid	description and use	reference	construct cargo present/absent in omv	
A.	pSB1A3	pSB1A3 is a high copy number plasmid carrying ampicillin resistance.The replication origin is a pUC19-derived pMB1 
	Registry of Biological Parts
IGEM
http://parts.igem.org/Main_Page 	ND	
B.	pSB1K3.m1	pSB1A3-1 is a high copy number plasmid carrying kanamycin resistance. 
	Registry of Biological Parts
IGEM
http://parts.igem.org/Main_Page 	ND	
C.	pSB1T3	pSB1A3-1 is a high copy number plasmid carrying tetracycline resistance. 
	Registry of Biological Parts
IGEM
http://parts.igem.org/Main_Page 	ND	
D.	pSB1C3	pSB1C3 is a high copy BioBrick assembly plasmid with a chloramphenicol resistance marker	Registry of Biological Parts
IGEM
http://parts.igem.org/Main_Page 	ND	
1	pSB1C3 gfp++.torA++
	Transportation of gfp in presence of torA signal peptide	For this research	+ve	
2	pSB1C3.gfp++.torA--
	Transportation of gfp in absence of torA signal peptide	For this research	-ve	
3	pSB1C3.torA
	Synthetic Biology plasmid containing torA signal peptide	For this research	+ve	
4	pSB1C3.Ail.torA
	Synthetic Biology plasmid containing torA signal peptide and invasive protein Ail from Yersinia pestis	For this research	+ve	
5	pSB1C3.Ail.gfp
	Synthetic Biology plasmid containing gfp and invasive protein Ail from Yersinia pestis	For this research	-ve	
6	pSB1C3.Ail.diINF-7.gfp.torA
	Synthetic Biology plasmid containing gfp and invasive construct  for Ail from Yersinia pestis along with fusogenic peptide from Influenza A virus (A/England/939/69(H3N2)) x (A/PR/8/34(H1N1)) along with torA construct	For this research	+ve	
7	pSB1C3.Ail.diINF-7.gfp.OmpA.torA
	Synthetic Biology plasmid containing gfp and invasive construct for Ail from Yersinia pestis along with fusogenic peptide construct from Influenza A virus (A/England/939/69(H3N2)) x (A/PR/8/34(H1N1)) along with torA and OmpA fragment construct to increase the invasiveness	For this research	+ve	
8	pSB1C3.Lux.torA
	Synthetic Biology plasmid containing luciferase cassette and periplasm targeting peptide fragment construct torA	For this research	+ve	
9	
pSB1C3.diINF-7
	Synthetic Biology plasmid containing fusogenic peptide fragment construct from Influenza A virus (A/England/939/69(H3N2)) x (A/PR/8/34(H1N1))	For this research	-ve	
10	pSB1C3.diINF-7.torA
	Synthetic Biology plasmid containing fusogenic peptide fragment construct from Influenza A virus (A/England/939/69(H3N2)) x (A/PR/8/34(H1N1)) along with periplasm targeting peptide fragment construct	For this research	+ve	
11	pSB1C3.diINF-7.Lux
	Synthetic Biology plasmid containing fusogenic peptide fragment construct from Influenza A virus (A/England/939/69(H3N2)) x (A/PR/8/34(H1N1)) and luciferase cassette	For this research		
12	pSB1C3.diINF-7.Lux.torA
	Synthetic Biology plasmid containing fusogenic peptide fragment construct  from Influenza A virus (A/England/939/69(H3N2)) x (A/PR/8/34(H1N1)) and luciferase cassette along with periplasm targeting peptide fragment construct	For this research	+ve	
13	pSB1C3.Ail.diINF-7.Lux.torA
	Synthetic Biology plasmid containing fusogenic peptide construct from Influenza A virus (A/England/939/69(H3N2)) x (A/PR/8/34(H1N1)) and luciferase cassette along with periplasm targeting peptide fragment and invasive construct for Ail 	For this research	+ve	
14	pSB1C3.Ail
	Synthetic Biology plasmid containing invasive construct for Ail	For this research	-ve	
15	pSB1C3.Ail.diINF-7.saporin
	Synthetic Biology plasmid containing fusogenic peptide construct from Influenza A virus (A/England/939/69(H3N2)) x (A/PR/8/34(H1N1)) along with ribosome inactivating protein construct from Saponaria officinalis and invasive construct for Ail	For this research	-ve	
16	pSB1C3.Ail.diINF-7.saporin.torA
	Synthetic Biology plasmid containing fusogenic peptide construct from Influenza A virus (A/England/939/69(H3N2)) x (A/PR/8/34(H1N1)) along with ribosome inactivating protein construct from Saponaria officinalis , invasive construct for Ail and torA	For this research	+ve	
17	pSB1C3.Ail.saporin
	Synthetic Biology plasmid containing ribosome inactivating protein construct from Saponaria officinalis and invasive construct for Ail	For this research	-ve	
18	pSB1C3.saporin.torA
	Synthetic Biology plasmid containing ribosome inactivating protein construct from Saponaria officinalis and periplasm directing construct torA	For this research	+ve	
19	pSB1C3.saporin	Synthetic Biology plasmid containing ribosome inactivating protein construct from Saponaria officinalis	For this research	-ve	
21	pSB1C3.Ail.diINF-7.torA
	Synthetic Biology plasmid containing invasive construct  for Ail from Yersinia pestis along with fusogenic peptide from Influenza A virus (A/England/939/69(H3N2)) x (A/PR/8/34(H1N1)) and torA construct for periplasmic transportation	For this research	+ve	
22	pSB1C3.Ail.diINF-7.p19.siRNA.torA
	Synthetic Biology plasmid containing invasive construct  for Ail from Yersinia pestis along with fusogenic peptide from Influenza A virus (A/England/939/69(H3N2)) x (A/PR/8/34(H1N1)) along with siRNA, ssRNA binding complex p19construct derived from Carnation Italian ringspot virus (CIRV)and torA construct for periplasmic transportation	For this research	+ve	
23	pSB1C3.Ail.diINF-7.siRNA.torA
	Synthetic Biology plasmid containing invasive construct  for Ail from Yersinia pestis and fusogenic peptide construct from Influenza A virus (A/England/939/69(H3N2)) x (A/PR/8/34(H1N1)) along with siRNA and torA construct for periplasmic transportation	For this research	+ve	
23	pSB1C3.p19.siRNA.torA
	Synthetic Biology plasmid containing invasive construct  for siRNA and ssRNA binding complex p19construct derived from Carnation Italian ringspot virus (CIRV) along with torA construct for periplasmic transportation	For this research	+ve	
24	pSB1C3.Ail.siRNA.torA
	Synthetic Biology plasmid containing invasive construct  for Ail from Yersinia pestis along with siRNA and torA construct for periplasmic transportation	For this research	+ve	
25	pSB1C3.siRNA.torA
	Synthetic Biology plasmid containing siRNA and torA construct for periplasmic transportation	For this research	+ve	


3.	Real time PCR primers
                             	Sequence
Probe                           5'-/56-FAM/CTG ATG GGC /ZEN/ACC TGG AGA GAG G/3IABkFQ/-3'
MYD88 forward         5'-CTG CAG AGC AAG GAA TGT GA-3'
MYD88 reverse          5'-TAG TCG CAG ACA GTG ATG AAC-3'
 â-actin forward      	5′-AGAGGGAAATCGTGCGTGAC-3′
â-actin reverse        	5′ CAATAGTGATGACCTGGCCGT-3′
Product Information
Assay Name: Hs.PT.56a.40601199.g Gene Name: MYD88
Product: PrimeTime® Std qPCR Assay RefSeqNumber: NM_002468
Probe Purification: HPLC Purification Exon Location: 5 - 6 

4.	siRNA designs
Construct myD88 #1   Position:1351
Sense1
5'ACCTCGATTATCAGCCAGGACACTATTCAAGAGATAGTGTCCTGGCTGATAATCTT3'
Antisense1
5'CAAAAAGATTATCAGCCAGGACACTATCTCTTGAATAGTGTCCTGGCTGATAATCG3'

Construct myD88 #2  Position:1623
Sense2
5'ACCTCGGCAGCTTCTTCCACAGTGATTCAAGAGATCACTGTGGAAGAAGCTGCCTT3'
Antisense2
5'CAAAAAGGCAGCTTCTTCCACAGTGATCTCTTGAATCACTGTGGAAGAAGCTGCCG3'
